# Latency-Associated Transcript-Derived MicroRNAs in Herpes Simplex Virus Type 1 Target SMAD3 and SMAD4 in TGF-β/Smad Signaling Pathway

**DOI:** 10.52547/ibj.25.3.169

**Published:** 2021-02-06

**Authors:** Zahra Shojaei Jeshvaghani, Ehsan Arefian, Sara Asgharpour, Masoud Soleimani

**Affiliations:** 1 Department of Biotechnology, College of Science, University of Tehran, Tehran, Iran;; 2 Department of Microbiology, School of Biology, College of Science, University of Tehran, Tehran, Iran;; 3 Stem Cell Technology Research Center, Tehran, Iran;; 4 Department of Hematology, Faculty of Medical Sciences, Tarbiat Modares University, Tehran, Iran

**Keywords:** Herpesvirus 1, Latency associated transcript, microRNA, SMAD3 protein, SMAD4 protein

## Abstract

**Background:**

During its latent infection, HSV-1 produces only a miRNA precursor called LAT, which encodes six distinct miRNAs. Recent studies have suggested that some of these miRNAs could target cellular mRNAs. One of the key cell signaling pathways that can be affected by HSV-1 is the TGF-β/Smad pathway. Herein, we investigated the potential role of the LAT as well as three LAT-derived miRNAs in targeting SMAD3 and SMAD4, as two main mediators in TGF-β/Smad.

**Methods:**

The selection of LAT-derived miRNAs was based on the search results obtained from an online miRNA prediction tool. HEK293T cells were transfected with each miRNA-expressing lentivector and with the construct-expressing LAT. To survey the effect of LAT on the expression of pro-fibrotic markers, we transfected LX-2 cells with LAT construct. The impact of viral miRNA overexpression on SMADs and fibrotic markers was measured by qPCR and luciferase assays.

**Results:**

Among the LAT-derived miRNAs, miR-H2, miR-H3, and miR-H4 were selected for the study. Our results demonstrated that while miR-H2 binds to both *SMAD* mRNAs, miR-H3 and miR-H4 inhibit only the expression of the *SMAD4* and *SMAD3*, respectively. Transfection of the LX-2 with LAT also decreased pro-fibrotic genes expression.

**Conclusion:**

Our findings display that LAT negatively regulates TGF-β/Smad through targeting *SMAD3* and *SMAD4* by its miRNAs. These viral miRNAs can also contribute to the development of therapeutic interventions in diseases for which prevention or treatment can be achieved through targeting TGF-β pathway.

## INTRODUCTION

Herpesviruses have been indicated to encode miRNAs^[^^1^^]^. Both viral and cellular targets have also been identified for miRNAs encoded by these types of viruses^[^^2^^]^. HSV-1 is a member of alphaherpesvirus subfamily and a ubiquitous human pathogen that causes most cold sores in humans. The virus primarily infects mucosal epithelium where it starts its productive cycle and then persists in the body by becoming latent and hiding from the immune system in the cell bodies of neurons in sensory ganglia. Through this stage, no viral protein is encoded, and no infectious virus is detected, but the virus can reactivate from latency and cause recurrent disease^[^^3^^]^. During latent infection, the only abundant viral gene product is a non-coding RNA, the LAT^[^^4^^]^. 

Up to now, several miRNAs, including HSV-1 miR-H1-H8^[^^5^^,^^6^^]^, miR-H11-H18^[^^7^^]^, and miR-H26-H29^[^^8^^-^^10^^]^, have been identified in HSV-1. Among them, six miRNAs, miR-H2-H5, -H7, and -H8, are within the second LAT exon and are typically expressed during latent infection. There are two miRNA pairs in HSV-1, miR-H1/miR-H6^[^^5^^]^ and miR-H2/miR-H14^[^^7^^]^, which are encoded on complementary strands. Since such pairs are transcribed from palindromic sequences, each pair shares a high sequence identity, suggesting that they might have conserved targets or prevent the transcription and/or function of their complementary miRNA. Interestingly, contrary to miRNAs originated from other HSV-1 genomic regions, those encoded within LAT all load into micro RISC (the key effector of miRNA function) with higher efficiencies and have highly conserved seed sequences, like most functional cellular miRNAs^[^^11^^]^. 

Viral mRNA targets have been identified for some HSV-1-encoded miRNAs like miR-H2-3p, which can decrease ICP0 protein expression^[^^5^^]^. ICP0 is a viral IE transcriptional activator and is important in HSV-1 replication and reactivation from latency^[^^12^^]^. miR-H6 can also inhibit the expression of another viral IE gene, ICP4^[^^5^^]^, which is critical for the expression of most HSV-1 genes in productive infection^[^^13^^]^. While both miR-H3 and miR-H4 originate from LAT sequences oriented antisense to the viral ICP34.5 mRNA, it is just miR-H4 that can bind to the mRNA and inhibit the expression of ICP34.5^[^^11^^]^. The ICP34.5 protein is a neurovirulence factor that plays critical roles in viral replication and anti-host responses^[^^14^^]^. So far, no cellular targets have been found for HSV-1 miRNAs except for mir-H27, which is thought to target mRNA of the cellular transcriptional repressor KLHL24 that reduces the transcriptional efficiency of viral IE and early genes^[^^9^^]^. Taken together, it seems necessary to make more effort towards the identification of cellular targets for miRNAs in HSV-1, and the specific mechanisms by which viral miRNAs accomplish their specific functions. In particular those LAT-encoded miRNAs that are homologous between the two stereotypes of HSV, may greatly influence the viral replication rate, load into the micro RISC with the highest efficiencies, and bear tightly conserved seed regions.

Previous studies have shown that HSV-1 infection can affect TGF-β pathway^[^^15^^-^^17^^]^. However, there is limited research on the mechanisms by which HSV-1 regulates this pathway. In general, signaling is initiated with ligand-induced oligomerization of the receptor kinases and phosphorylation of the R-SMADs, which include SMAD2 and SMAD3 for the TGF-β/Smad pathway. Activated R-SMADs partner with the common signaling transducer SMAD4 (Co-SMAD) and together travel to the nucleus where they affect transcription factors. In normal states, TGF-β pathway has an important function in the maintenance of homeostasis, while it is chronically over-activated in some diseases, including cancer, inflammation, and tissue (renal, hepatic, and pulmonary) fibrosis^[^^18^^-^^20^^]^.

Whether any of miRNAs originated from LAT region can play a role in the down-regulation of TGF-β/Smad signaling is currently unknown. Here, we determined the inhibitory effect of whole LAT sequence and LAT-derived miRNAs in HEK293T cells on the expression of SMAD3 and SMAD4, as important intermediate proteins in the Smad pathway. To test the effect of LAT on a state caused by the stimulation of TGF-β/Smad pathway, we employed activated LX-2 cells and found that LAT transcript can negatively regulate the expression of pro-fibrotic markers in these cells, which can be a consequence of the TGF-β/Smad subsidence through targeting SMAD3 and SMAD4 by LAT miRNAs. 

## MATERIALS AND METHODS

**Bioinformatics analysis**

Sequences of LAT miRNAs in HSV-1— miR-H2 (MI0008937), miR-H3 (MI0008938), miR-H4 (MI0008939), and miR-H5 (MI0008940)— were obtained from the miRBase database (release 22; http://www.mirbase.org/). Using the TargetScan Custom (release 5.2; http://www.targetscan.org/ vert_50/seedmatch.html), targeting SMAD3 (NM_001145102), and SMAD4 (NM_005359) mRNAs were estimated for each LAT-derived miRNA. 

**Construction of recombinant plasmids**

**Fig. 1:**
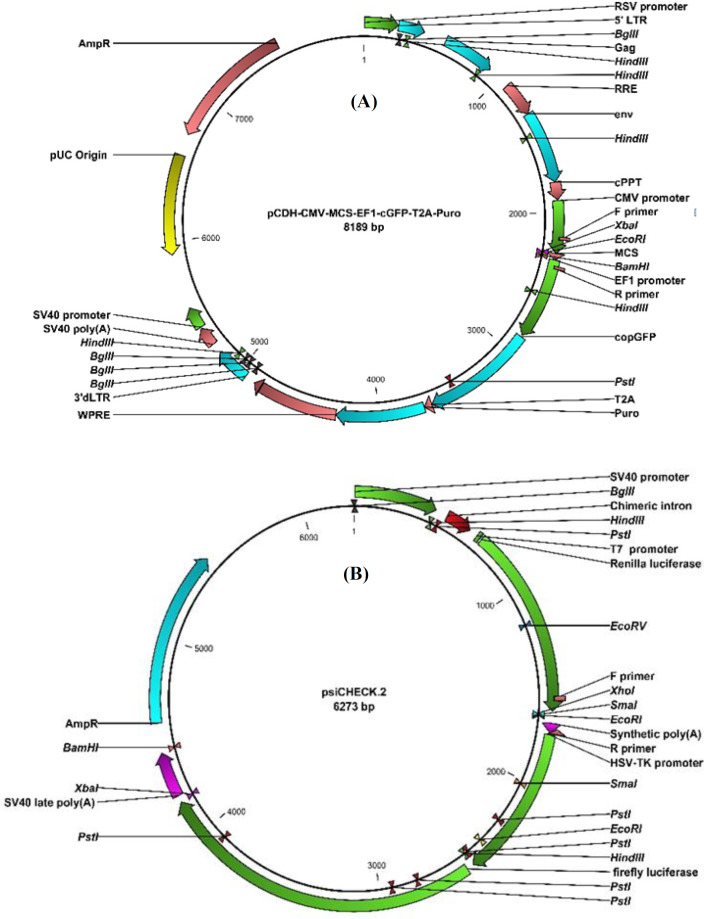
Vectors used in the construction of recombinant plasmids. (A) The Lentiviral pCDH vector contains common domains such as selection antibiotics, copGFP expression cassette under the control of the EF1βα promoter, and the cytomegalovirus (CMV) promoter that leads to the production of our selected viral miRNAs inserted between the *Eco*RI and *Bam*HI sites. (B) psiCHECK-2 vector designed to provide quantitative and rapid optimization of RNA interference. Renilla luciferase was used as the primary reporter gene, and the 3’-UTR of SMAD3 and SMAD4 was separately cloned into the *Xho*I and *Not*I sites located downstream of the Renilla translational stop codon. Introduction of firefly luciferase in this vector, as a second reporter gene, allows the normaliztion of Renilla luciferase expression, achieving robust and reproducible results

Primary sequences of HSV-1 miR-H2, miR-H3, and miR-H4 were separately synthesized in pUC57 vector (GenScript, NJ, USA). Using the restriction enzymes *Bam*HI And *Eco*RI (Thermo Fisher Scientific Inc., Massachusetts, USA), each miRNA was then inserted into pCDH-CMV-MCS-EF1-cGFP-T2A-Puro vector (System Biosciences, California, USA), which is herein termed pCDH (Fig. 1). pcDNA3(-)-LAT construct was kindly donated by Professor Bryan R. Cullen from Duke University Medical Center (USA). To construct 3’-UTR reporter plasmids, the 3’-UTR fragments of human SMAD3 and SMAD4 mRNAs containing the putative miRNA binding sites were amplified from human genomic DNA using the designed primers (SMAD3 F: 5’ -CAC CTC GAG TGG ATT GAG CTG CAC CTG- 3’ R: 5’ -TAG CGG CCG CAT CAC CAG AAT CAG GCT GTG- 3’; SMAD4 F: 5’ -TCG ACT CGA GAA CCT GCC TTG TGG GAT TTG- 3’ R: 5’ -GGC CGC GGC CGC TGC CTC TGT CTT CAC ATC GC- 3’. Each fragment was individually cloned into the *Xho*I and *Not*I sites of the luciferase vector psiCHECK-2 (Promega, USA; Fig. 1). Restriction enzyme digestion and the sequencing method confirmed that both viral miRNAs and 3’-UTR fragments have been accurately inserted into the vectors.

**Cell lines and cell transfection**

This study employed HEK293T cells (donated by Stem Cell Technology Research Center, Tehran, Iran) and the human HSC line, LX-2 (donated by Dr. Seyed Younes Hosseini, Shiraz University of Medical Sciences, Shiraz, Iran), which provides a valuable new tool in the *in vitro* studies of liver diseases such as liver fibrosis. LX-2 cells are viable in serum-free media and highly transfectable^[^^21^^]^. Both cell lines were maintained in cell culture flasks in high glucose DMEM (Sigma Chemical Co., St. Louis, MO, USA) supplemented with 10% FBS (Sigma Chemical Co.) for HEK293T and 5-7% FBS plus TGF-β1 (5 ng/mL; PeproTech, London, UK) for LX-2 cells. Twenty-four hours before transfection, cells were seeded into 12-well cell culture plates, such that they will be 70-90% confluent at the time of transfection, and were incubated in a 37 °C incubator with 5% CO_2_. Before transfection, the culture medium was replaced with antibiotics-free DMEM supplemented with 10% FBS for HEK293T and 2% FBS plus TGF-β1 (5 ng/mL) for LX-2 cells. The plasmids expressing viral miRNAs, LAT, and the control vector were independently transfected into the cells using Lipofectamine 2000 (Invitrogen, USA) according to the manufacturer’s instructions. 

**qRT-PCR for miRNAs and mRNAs**

Total RNAs were extracted from cells with TRIzol reagent (Invitrogen) 48 h after transfection. The relative expression of miRNAs was measured using quantitative stem-loop reverse transcription-PCR. The qPCR reactions were performed using qPCR Master Mix 2× High Rox (Ampliqon, Denmark) and a set of miRNA-specific probes and primers (Table 1) in a TaqMan probe-based fashion. SNORD47 was used as an endogenous control for the normalization of the expression levels. For mRNA quantification, cDNAs were generated using a cDNA synthesis kit (Thermo Fisher Scientific Inc.), and qPCR was performed using SYBR Green qPCR Master Mix 2× Hhigh Rox (Ampliqon) and designed primer sequences (Table 2). Expression of mRNAs was normalized against B2M. RT-PCR was performed on an ABI Prism 7500 RT-PCR system, and the expressions of miRNAs and mRNAs were quantified by measuring Ct values using the 2^-ΔΔCt^ method in REST 2009 software.

**Table 1:** Primers and TaqMan probes used in qRT-PCR for miRNAs

**Small RNA**	**Set of miRNA-specific probes and primers**
miR-H2-3p	RT: 5’-GTC GTA TGC AGA GCA GGG TCC GAG GTA TTG GCA CTG CAT ACG ACA GTC GC-3’ Forward: 5’-CTG AGC CAG GGA CGA GT-3’ Probe: 5’-FAM-CGA CTG TCG TAT GCA GTG CC-BHQ-1-3’
miR-H3-3p	RT: 5’-GTC GTA TGC AGA GCA GGG TCC GAG GTA TTG GCA CTG CAT ACG ACG TCC CA-3’ Forward: 5’-CCA CTG GGA CTG TGC G-3’ Probe: 5’-FAM-TGG GAC GTC GTA TGC AGT GC-BHQ-1-3’
miR-H4-3p	RT: 5’-GTC GTA TGC AGA GCA GGG TCC GAG GTA TTC GCA CTG CAT ACG ACA CTA GC-3’ Forward: 5’-AGC CCT TGC CTG TCT AAC-3’ Probe: 5’-FAM-GCC TAG TGT CGT ATG CAG TGC-BHQ-1-3’
miR-H4-5p	RT: 5’-GTC GTA TGC AGA GCA GGG TCC GAG GTA TTC GCA CTG CAT ACG ACT GCT GC-3’ Forward: 5’-CGC AGG TAG AGT TTG ACA G-3’ Probe: 5’-FAM-CAA GCA GTC GTA TGC AGT GCG-BHQ-1-3’
miR-H5-3p	RT: 5’-GTC GTA TGC AGA GCA GGG TCC GAG GTA TTC GCA CTG CAT ACG ACC CGG AG-3’ Forward: 5’-GTC GTC AGA GAT CCA AAC C-3’ Probe: 5’-FAM-TCC GGG TCG TAT GCA GTG C-BHQ-1-3’
miR-H7	RT: 5’-CTC GTA TGC AGA GCA GGG TCC GAG GTA TTC GCA CTG CAT ACG AGC CTT TG-3’ Forward: 5’-CAC AAA GGG GTC TGC AA-3’ Probe: 5’-FAM-CAA AGG GTC GTA TGC AGT GC-BHQ-1-3’
miR-H8	RT: 5’-GTC GTA TGC AGA GCA GGG TCC GAG GTA TTC GCA CTG CAT ACG ACG AAC CC-3’ Forward: 5’-GAT GCG TAT ATA GGGT TCA GG-3’ Probe: 5’-FAM-GGT TCG TCG TAT GCA GTG CG-BHQ-1-3’
SNORD47	RT: 5’-GTC GTA TGC AGA GCA GGG TCC GAG GTA TTC GCA CTG CAT ACG ACA ACC TC-3’ Forward: 5’-ATC ACT GTA AAA CCG TTC CA-3’ Probe: 5’-FAM-TGA TTC TGA GGT TGT CGT ATG CA-BHQ-1-3’

RT, reverse transcription primer

**Table 2:** Primers used in qRT-PCR for mRNAs

** Gene**	**Product size (bp)**	**Primer sequence **
*B2M*	91	F: 5’-ATG CCT GCC GTG TGA AC-3’ R: 5’-ATC TTC AAA CCT CCA TGA TG-3’
*MAPK1*	81	F: 5’-CAT GGT GTG CTC TGC TTA TG-3’ R: 5’-GTA GGT CTG GTG CTC AAA GG-3’
*SMAD2*	124	F: 5’-AGC AGA ATA CCG AAG GCA GAC G-3’ R: 5’-TTG AGC AAC GCA CTG AAG GG-3’
*SMAD3*	186	F: 5’-CGG AGA CAC ATC GGA AGA G-3’ R: 5’-CGA ACT CCT GGT TGT TGA AG-3’
*SMAD4*	163	F: 5’-CCA ACT TTC CCA ACA TTC C-3’ R: 5’-GGT AGT GCT GTT ATG ATG GTA AG-3’
*Col I*	121	F: 5’-TGG AGC AAG AGG CGA GAG-3’ R: 5’-CAC CAG CAT CAC CCT TAG C-3’
*Col II*	85	F: 5’-GGT CTT GGT GGA AAC TTT GCT-3’ R: 5’-GGT CCT TGC ATT ACT CCC AAC-3’
*Col III*	158	F: 5’-CCA GGT GCT GAT GGT GTC-3’ R: 5’-ACC TCT CTC ACC AGG GCT-3’
*MMP2*	136	F: 5’-GCT CGT GCC TTC CAA GTC-3’ R: 5’-AGT CCG TCC TTA CCG TCA-3’

F, forward; R, reverse

**MTT assay**

Cells were cultured on three 96-well plates at a density of 4 × 10^4^ cells/well and then independently transfected with recombinant constructs, each expressing miR-H2, miR-H3, miR-H4, LAT, and control vector in triplicate. At 24 h, 48 h, and 72 h after transfection, cells were incubated with 100 µl of MTT assay reagents 1× (Sigma-Aldrich, USA) in a 37 °C incubator with 5% CO_2 _for 3 h. The absorbance at 570 nm was measured for both transfected and un-transfected cells using an Absorbance Microplate Reader (BioTek™ ELx800™, USA). 

**Luciferase assay**

Luciferase reporter assays were performed using the dual luciferase reporter assay system (Promega) according to the manufacturer’s instructions. Both constructed psiCHECK2-SMAD3 and psiCHECK2-SMAD4 were separately co-transfected with pCDH-miR-H2, pCDH-miR-H3, pCDH-miR-H4, pcDNA3(-)-LAT, or control vector into HEK293T cells. Luciferase activity was measured using a SIRIUS Luminometer (Berthold Detection Systems GmbH, Germany) in cell lysates 48 hours after transfection. Results were normalized against firefly luciferase activity.

**Statistical analysis**

Data were expressed as mean ± SD of at least three independent experiments. Student’s unpaired *t*-tests were used to perform statistical analysis in GraphPad Prism6. *p* < 0.05 was considered statistically significant. 

## RESULTS

***Selection of LAT-encoded miRNAs for experimental evaluation***

**Fig. 2:**
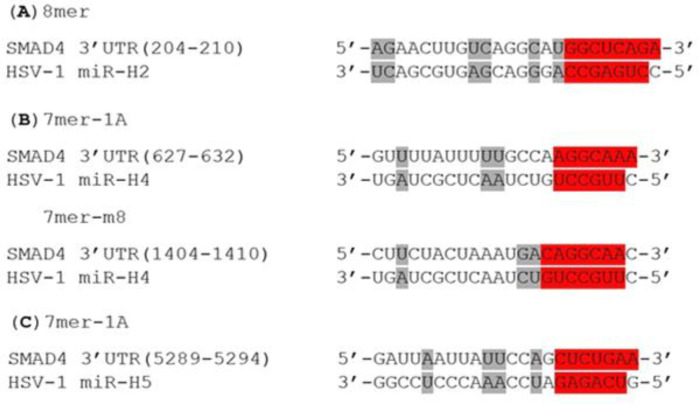
Bioinformatic prediction of targeting SMAD4 by LAT-derived miRNAs (A) miR-H2, (B) miR-H4, and (C) miR-H5 target SMAD4 3’UTR at different sites. Predicted binding site types are indicated in red, and other complementary pairing bases are shown in gray. TargetScan Custom defines site types as below: 8mer: an exact match to positions 2-8 of the mature miRNA, followed by an 'A'; 7mer-m8: an exact match to positions 2-8 of the mature miRNA; 7mer-1A: an exact match to positions 2-7 of the mature miRNA, followed by an 'A'

In order to select the most promising LAT miRNAs for investigation, we employed an established online miRNA prediction software, TargetScan Custom (release 5.2; http://www.targetscan.org/vert_50/ seedmatch.html). This program forecasts biological targets of assigned miRNAs by searching for the presence of 8mer and 7mer evolutionary conserved sites that match the seed region (positions 2-7 at the 5’ end of the mature miRNAs) of each miRNA. According to this analysis, none of the LAT miRNAs were predicted to target SMAD3, while miR-H2 (with the best site type), H4-3p (with two sites), and H5-3p were likely to bind to SMAD4 mRNA (Fig. 2). Another factor we have taken into account is the fact that HSV-1 miRNAs are loaded into the RISC with different efficiencies^[^^11^^]^. Overall, considering the bioinformatic predictions and the above-mentioned report, we selected miR-H2, miR-H3, and miR-H4 for the investigation of their potential in targeting SMAD3 and SMAD4 mRNAs.

**Induction of miRNAs in transfected cells**

**Fig. 3:**
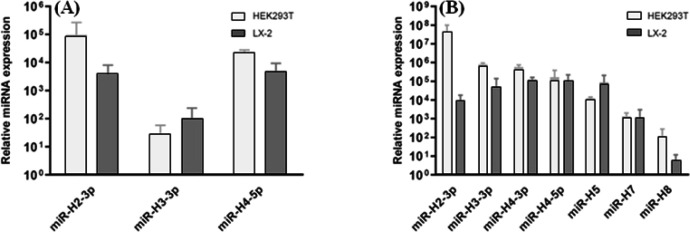
qRT- PCR analysis of miRNAs expression in transfected cells. (A) Overexpression of selected miRNAs in both T293 and LX-2 cells, 48 h after transfection with the recombinant construct expressing miR-H2, -H3, or -H4 against control group transfected with empty pCDH. (B) Relative expression of LAT-derived miRNAs in HEK293T and LX-2 cells transfected with pcDNA3-LAT compared to cells transfected with control vector, pcDNA3(-), in 48 h. Relative levels of miRNA expression were normalized to SNORD47. Data were reported as mean ± SD (n = 3; *p* < 0.001)

Transfection of both HEK293T and LX-2 cells with any of our recombinant plasmids resulted in the induction of the subjected miRNA in comparison to the control group, indicating our constructs succeeded to overexpress miRNAs sufficiently (Fig. 3A). This upregulation provided us with the cells in which we could evaluate the effect of selected miRNAs on the expression of target genes. In addition, qRT-PCR analysis verified the presence of all six LAT-derived miRNAs, in both HEK293T and LX-2 cells transfected with pcDNA3-LAT against the control group (Fig. 3B). miR-H2 was the most abundant miRNA in 293T, and miR-H4 and miR-H5 had the most abundances in LX-2. Although detected, miR-H8 has been found to be present at very low levels, specifically in LX-2, in comparison to other miRNAs, which is compatible with a previous report on HSV-1 miRNAs expression profiles in infected cells^[^^6^^]^. 

**Effect of LAT-derived miRNAs on cell viability in** **HEK** **293T and LX-2 cells**

**Fig. 4:**
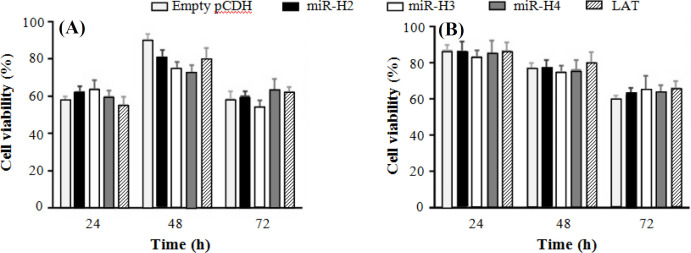
Cell viability of transfected cells. MTT assay was performed on HEK293T (A) and LX-2 (B) cells post 24 h, 48 h, and 72 h of transfection with miR-H2, miR-H3, miR-H4, LAT, or empty pCDH as a control vector. The histogram shows the mean values of cell viability percentage against untransfected cells from three independent experiments

The results from MTT assay confirmed that in comparison to the control groups transfected with control vector, neither LAT nor any of LAT-derived miRNAs studied here (miR-H2, -H3, and -H4) could significantly influence cell viability in both HEK293T and LX-2 cells. General low cell viability, especially 24 h after transfection in HEK293T cells, was probably due to the severe toxicity commonly observed with Lipofectamine 2000^[^^22^^]^. Compared to the control group, lower viability was only observed in HEK293T cells 48 h after transfection with viral miRNAs. Nevertheless, there was no considerable difference in the HEK293T viability of different groups 72 h after transfection, indicating that viral miRNAs did not possess cytotoxic effect on the cells (Fig. 4).

**Direct targeting of SMAD3 and SMAD4 by LAT-derived miRNAs**

**Fig. 5:**
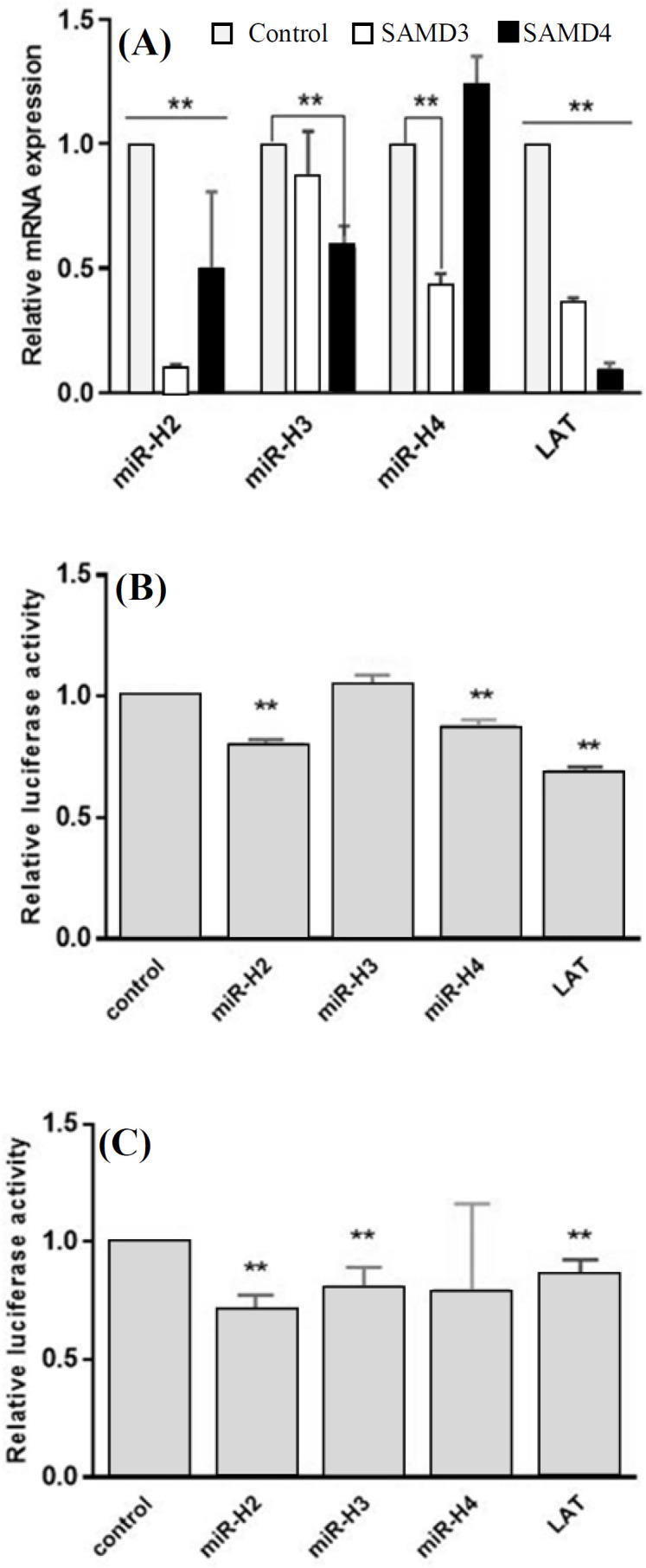
SMAD3 and SMAD4 as primary targets for LAT-derived miRNAs. (A) HEK293TqRT-PCR analysis of SMAD3 and SMAD4 in HEK293T cells transfected with pCDH-miR-H2, pCDH-mir-H3, pCDH-miR-H4, or pcDNA3-LAT against cells transfected with empty pCDH or pcDNA3(-) as control vectors. Relative levels of gene expression were normalized to B2M mRNA level. ^**^p < 0.001; Luciferase activity assay for direct targeting of the 3’-UTR of (B) SMAD3 and (C) SMAD4 by miR-H2, miR-H3, mir-H4, or LAT. Partial segments of SMAD3 and SMAD4 were fused with a luciferase reporter and co-transfected into HEK293T cells with the recombinant plasmid containing miR-H2, -H3, -H4, LAT, or empty pCDH. Luciferase activity was measured 48 h post transfection. Levels of Renilla luciferase activity were normalized to firefly luciferase activity as an internal control. ^**^p < 0.0001

To test whether our selected miRNAs inhibit the expression of SMAD3 and SMAD4, we quantified the relative expression of these genes in HEK293T cells separately transfected with pcDNA3-LAT and also our recombinant constructs, each expressing miR-H2, miR-H3, or miR-H4. According to qRT-PCR results, LAT decreased the expression level of both genes. Among miRNAs, HSV-1 miR-H2 also negatively regulated both targets, though SMAD3 was not predicted as a target for it. On the other hand, miR-H3 and miR-H4 downregulated only SMAD4 and SMAD3, respectively. This result is contrary to the computational analysis by which miR-H3 was predicted to target neither SMAD3 nor SMAD4, and miR-H4 was conversely supposed to bind to SMAD4 mRNA (Fig. 5A). In order to investigate whether SMADs mRNA are the primary target of miRNAs or not, the ability of each miRNA to regulate the 3’UTR region of SMAD3 and SMAD4 mRNAs was evaluated via dual-luciferase reporter assay. Human HEK293T cells were used to verify this effect. There was a perfect base pairing between the seed sequence of selected miRNAs (miR-H2 and -H4) and the 3’-UTR of SMAD4 mRNA (Fig. 2). The 3’UTR target sites of SMAD4 and a partial segment of SMAD3 3’UTR were cloned into the psiCHECK-2 vector (Fig. 1), downstream of a Renilla luciferase. The addition of pcDNA3-LAT plasmid, but not the control vector, significantly suppressed the luciferase activity upon co-transfection of the luciferase vectors containing the 3’UTR target sites of SMAD4 or partial segment of SMAD3 3’UTR (Fig. 5B and C). In agreement with qRT-PCR results, the luciferase assay confirmed that miR-H2 and miR-H4 specifically bound SMAD3 (Fig. 5B). SMAD4 was also a direct target for miR-H2 and miR-H3 (Fig. 5C). The results demonstrated that LAT performed its inhibitory effect on SMAD3 and SMAD4 genes by these miRNAs.

**LAT suppressed pro-fibrotic markers in LX-2 cells**

**Fig. 6:**
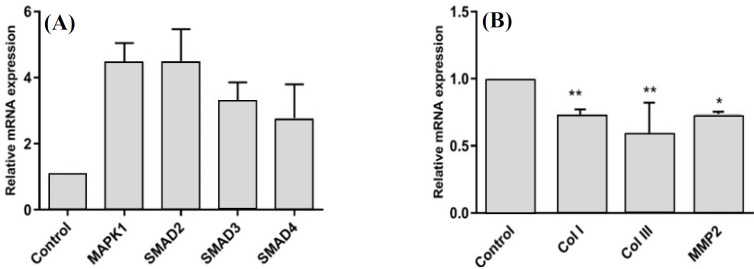
qRT- PCR analysis of mRNAs expression in the activated HSC, LX-2. (A) Upregulation of TGF-β/Smad signaling genes in LX-2 cells under the treatment of TGF-β1 (5 ng/ml) for 48 h in comparison to the untreated control group (*p* < 0.0001). (B) Downregulation of fibrogenic genes in TGF-β1treated cells 48 h post transfection with pcDNA3-LAT against treated cells transfected with empty pcDNA3(-) (^*^p < 0.05 and ^**^p < 0.001). Relative levels of gene expression were normalized to B2M mRNA level

Immortalized human HSC, LX-2, are classified as an activated phenotype expressing mRNAs for fibrogenic molecules and pathways of human hepatic fibrosis, including GF-β/Smad. We observed that the treatment of LX-2 cells with TGF-β1 (5 ng/ml) led to the increased mRNA expression of genes involved in the TGF-β/Smad pathway (Fig. 6A). Therefore, we employed LX-2 cells activated with TGF-β1, as an appropriate cell line for examining the suppressive effect of LAT on fibrogenic behavior, which is a phenotype originated from TGF-β pathway activation. In order to study this effect, we examined the relative expression of some fibrogenic markers in activated LX-2 cells, which were transfected with pcDNA3- LAT (Fig. 6B). 

## DISCUSSION

The SMAD proteins are nuclear effectors of TGF-β and can regulate transcription in a cell-type-specific and ligand dose-dependent fashion through interaction with transcription factors, coactivators, and corepressors. SMAD3 and SMAD4 exhibit that inherent sequence-specific DNA binding activity is probably involved in nuclear shuttling, although their affinity for DNA is not considerable^[^^23^^]^. It has been demonstrated that in viral infections, TGF-β expression can be modulated by SMAD proteins. Collectively, reports have suggested that in viral infections, the suppression of TGF-β/Smad signaling could contribute to the carcinogenesis. For example, in cells infected with human papillomavirus, viral oncoprotein E7 interacts constitutively with SMAD2, SMAD3, and SMAD4 and then occludes both SMAD transcriptional activity and the ability of TGF-β to prevent DNA synthesis^[^^24^^]^. In Kaposi's sarcoma-associated herpes virus infection, viral IFN regulatory factor 1 inhibits TGF-β pathway via its direct interaction with both SMADad3 and SMAD4 and through the prevention of their transactivation activity^[^^25^^]^. In adult T-cell leukemia cells infected with HTLV-1, virus oncoprotein Tax inhibits TGF-β signaling by reducing the SMAD3 DNA binding activity^[^^26^^]^. The DNA-binding activity of SMAD3/4 transcription factors complex is inhibited by tumor-derived hepatitis C virus core variants through a direct interaction between the central domain of tumor-derived core and the MH1 DNA-binding domain of SMAD3^[^^27^^]^. HSV-1 infection in human corneal epithelial cells causes a decline in TGF-β1 and SMAD3 expression but has no effect on the expression of SMAD2^[^^16^^]^. In a study by Gupta *et al.*^[^^15^^]^, it has been claimed that a miRNA generated from the HSV-1 LAT gene performs its anti-apoptotic effect by downregulating TGF-β1 and SMAD3 expression. Although there was controversy over the detection of the miRNA, the inhibitory effect of HSV-1 on TGF-β mRNA expression remained verified. Given the fact that LAT is the only viral gene expressed during latent infection in neurons, functioning as a primary miRNA precursor for encoding more than 50% of functionally important HSV-1 miRNAs, we supposed that LAT transcript could be responsible for the inhibition of SMAD-mediated TGF-β signaling observed in HSV-1-infected cells.

The goal of the present study was to determine which of the six miRNAs encoded within LAT in HSV-1 could target SMAD3 and SMAD4, as two important functionally linked mediators in TGF-β/Smad signaling pathway. Based on a study by Flores *et al.*^[^^11^^]^, miR-H2, miR-H3, and miR-H4 are three of the most highly expressed HSV-1 miRNAs and have the most reads in deep sequencing of RISC-associated small RNAs, implying their functionality. On the other hand, while miR-H2 and miR-H4 target viral mRNAs, no viral target could be detected for miR-H3, suggesting that this viral miRNA could perform its function through binding to cellular mRNAs as previously reported for several other herpesvirus miRNAs^[^^28^^]^. 

Herein, we selected miR-H2, miR-H3, and miR-H4, based on computational analysis for the complementary base pairing of each LAT-encoded miRNA with SMAD mRNAs in a seed sequence-specific manner. We also took advantage of the results from previous studies and selected those miRNAs that are highly expressed at both productively and latently infected cells and are loaded into the RISC with the most efficiencies among other miRNAs in LAT^[^^5^^,^^6^^,^^11^^]^. Our qRT-PCR and luciferase activity results indicated that the LAT sequence significantly inhibited the expression of SMAD3 and SMAD4 mRNAs in HEK293T cells. A large number of studies have represented that the dysregulation of TGF-β/Smad pathway is an important pathogenic mechanism in tissue fibrosis^[^^29^^]^. Their viability in serum-free media and high transfectability have made LX-2 cells worthy models for *in vitro* studies of liver fibrosis^[^^21^^]^. LX-2 cells are reactive to TGF-β1, a major fibrogenic cytokine in liver disease. TGF-β1 directly activates Smad signaling, which triggers pro-fibrotic gene overexpression. TGF-β1 is increased in animal models of liver fibrosis and in patients with chronic liver disease^[^^30^^]^. The qRT-PCR results of this study also highlighted a considerable effect of LAT induction on the downregulation of profibrotic genes in activated LX-2 cells, an observation that we assume was the consequence of Smad signaling adjustment by LAT-derived miRNAs. Among miRNAs originated from LAT, we found that miR-H3-3p and miR-H4-3p could directly target SMAD4 and SMAD3 mRNAs, respectively, while miR-H2-3p bound to both SMAD mRNAs in their 3’UTR region and decreased their mRNA expression. Surprisingly, there was not a general agreement between our results and what has been predicted by TargetScan Custom, emphasizing that bioinformatic predictions are required to become necessarily approved by experimental validation. This inconsistency might be because of the fact that multiple potential miRNA recognition sites are identified in mRNAs, computationally, while in practice, miRNAs interact with their targets in complex ways. On the other hand, in TargetScan Custom, sites with poor seed pairing are excluded, resulting in some targets to be neglected. Whether or not other miRNAs originated from LAT region could regulate SMAD3 and SMAD4 needs to be studied. 

A decline in SMAD3 and SMAD4 expression could obviously reduce the formation and nuclear import of transcriptionally active SMAD heterotrimeric or dimeric complexes, giving rise to the decreased transcription of TGF-β1-regulated target genes. For the first time, we found cellular targets in TGF-β/Smad signaling for HSV-1 LAT-derived miRNAs in this study. Our results suggest that SMAD3 and SMAD4 may be involved in the pathology of diseases associated with HSV-1 infection such as cold sores. Although no function has been definitively shown for LAT, it has been suggested to be involved in heterochromatin assembly, restriction of accumulation of productive cycle gene products, prevention of cellular apoptosis in response to infection, latency establishment, and even reactivation from latency, all by unknown mechanisms^[^^6^^,^^31^^-^^33^^]^. It can be speculated that LAT-encoded miRNAs modulate Smad signaling by targeting SMAD3 and SMAD4 to prevent apoptosis in infected cells and then contribute to the persistence of HSV-1 through latent infection. However, the specific function of SMADd3 and SMAD4 in HSV-1 infection needs additional examination.

Last but not least, the administration of TGF-β antagonists, including soluble receptors, antibody, small molecular inhibitors, and mi/siRNAs that specifically target TGF-β1/Smad signaling, may therapeutically intervene the fibrosis of various tissues^[^^29^^,^^34^^]^. Our findings not only help to explore virus-host interactions, especially during latent HSV-1 infection, but they can also contribute to the development of therapeutic strategies in diseases in which the blockade of TGF-β signaling pathway by antagonists such as miRNAs leads to prevention or treatment. 
